# Identification of a recombinant equine coronavirus in donkey, China

**DOI:** 10.1080/22221751.2022.2056522

**Published:** 2022-04-04

**Authors:** Peng-Fei Qi, Xing-Yi Gao, Jing-Kai Ji, Yan Zhang, Shao-Hua Yang, Kai-Hui Cheng, Ning Cui, Man-Ling Zhu, Tao Hu, Xuan Dong, Bin Yan, Chang-Fa Wang, Hong-Jun Yang, Wei-Feng Shi, Wei Zhang

**Affiliations:** a Key Laboratory of Livestock and Poultry Multi-omics of MARA, and Shandong Key Laboratory of Animal Disease Control and Breeding, Institute of Animal Science and Veterinary Medicine, Shandong Academy of Agricultural Sciences, Ji’nan, People’s Republic of China; b School of Medicine, Shandong University of Traditional Chinese Medicine, Ji’nan, People’s Republic of China; c School of Life Sciences, Shandong First Medical University & Shandong Academy of Medical Sciences, Taian, People’s Republic of China; d Institute of Crop Germplasm Resources, Shandong Academy of Agricultural Sciences, Ji’nan, People’s Republic of China; e School of Public Health, Shandong First Medical University & Shandong Academy of Medical Sciences, Taian, People’s Republic of China; f Yellow Sea Fisheries Research Institute, Chinese Academy of Fishery Sciences, Qingdao, People’s Republic of China; g Liaocheng Research Institute of Donkey High-Efficiency Breeding and Ecological Feeding, Liaocheng University, Liaocheng, People’s Republic of China

**Keywords:** Equine coronavirus, recombination, donkey, bioinformatics analyses, China

## Abstract

Equine coronavirus (ECoV) was first identified in the USA and has been previously described in several countries. In order to test the presence of ECoV in China, we collected 51 small intestinal samples from donkey foals with diarrhoea from a donkey farm in Shandong Province, China between August 2020 and April 2021. Two samples tested positive for ECoV and full-length genome sequences were successfully obtained using next-generation sequencing, one of which was further confirmed by Sanger sequencing. The two strains shared 100% sequence identity at the scale of whole genome. Bioinformatics analyses further showed that the two Chinese strains represent a novel genetic variant of ECoV and shared the highest sequence identity of 97.05% with the first identified ECoV strain – NC99. In addition, it may be a recombinant, with the recombination region around the NS2 gene. To our knowledge, this is the first documented report of ECoV in China, highlighting its risk to horse/donkey breeding. In addition, its potential risk to public health also warrants further investigation.

Coronaviruses are enveloped ss+RNA viruses, currently including four genera: *Alphacoronavirus*, *Betacoronavirus*, *Gammacoronavirus*, and *Deltacoronavirus*. The genus *Betacoronavirus* consists of five subgenera: *Embecovirus*, *Hibecovirus*, *Merbecovirus*, *Nobecovirus*, and *Sarbecovirus* [1]. There are a few human coronaviruses in the genus *Betacoronavirus*, including severe acute respiratory syndrome coronavirus (SARS-CoV) and SARS-CoV-2 [2] in the subgenus *Sarbecovirus*, and Middle East respiratory syndrome coronavirus (MERS-CoV) in the subgenus *Merbecovirus*. The identification of close relatives of human coronaviruses from multiple wild animals suggested both the wide host spectrum of human coronaviruses and their animal origins [3]. In particular, genetic recombination has been found pervasive in betacoronaviruses, which increases the genetic diversity and may also be involved in the origins of several human coronaviruses [4]. For example, SARS-CoV might have originated from bat coronaviruses via multiple recombination events [5], and MERS-CoV might be also derived from recombination events among bat and camel coronaviruses [6].

The subgenus *Embecovirus* currently contains five species: Betacoronavirus 1, China Rattus coronavirus HKU24, Human coronavirus HKU1, Murine coronavirus, and Myodes coronavirus 2JL24. Equine coronavirus (ECoV) is classified as a member of the species Betacoronavirus 1 along with human coronavirus OC43, bovine coronavirus, canine respiratory coronavirus, porcine haemagglutinating encephalomyelitis virus (PHEV), and a few camel coronaviruses. The main clinical signs of ECoV infection in adult horses include anorexia, lethargy, and fever. Some infected individuals may develop diarrhoea and mild colic signs, although less frequently. The first ECoV strain, NC99, was isolated from a diarrhoeic foal in the USA, and it was subsequently identified in Japan, France, Saudi Arabia, United Kingdom, and Ireland in foals, adult horses, and donkeys with respiratory diseases and diarrhoea, respectively [7–9]. In particular, the seroprevalence of ECoV in horses in Israel reached 12.3%, comparable to that in the USA (9.6%) [10], highlighting its potential risk to horse husbandry. However, none ECoV infection has been documented in China.

In this study, a total of 51 small intestinal samples from donkey foals with diarrhoea were collected from a donkey farm in Shandong Province, China between August 2020 and April 2021. All samples were collected by dissection after the animals were killed, which were stored in liquid nitrogen and transported to our laboratory for analysis.

A real-time quantitative PCR (RT-qPCR) assay was established targeting a specific gene region of 142 nt in length in the nucleocapsid (N) gene of ECoV and was performed as previously described [11]. Two samples, 2020/7881 collected in November 2020 and 2021/464693 collected in April 2021 tested positive for ECoV, with cycle threshold (*Ct*) values of 17.43 and 18.96, respectively, while *Ct* values of the remaining samples were greater than 35.

The two RT-qPCR positive samples were homogenated as a 10% suspension in RPMI1640 and filtrated with a 0.22 μm filter. The supernatant was inoculated onto confluent monolayers of human rectal adenocarcinoma (HRT-18G) cells in 6-well plates and incubated for 90 min at 37°C under a 5% CO2 atmosphere for adsorption. After washing twice with phosphate-buffered saline (PBS), 2 ml fresh RPMI1640 medium with 0.25 μg/ml TPCK trypsin were added as maintenance medium. After six days, the culture supernatant was inoculated with fresh HRT-18G cells. After three passages at six-day-intervals, RT-qPCR test for ECoV was performed, with *Ct* value of 16.71 for 2021/464693 and 36.89 for 2020/7881. Transmission electron microscope examination was then operated for the cultured cells of ECoV_2021/464693. We observed several spherical virus particles with some pleomorphism inside the cells, and the virus particles were ∼100 nm in diameter with spikes (Figure S1), whose morphology is consistent with the *Coronaviridae* family.

Next-generation sequencing (NGS) was then performed to obtain the full-length genome of the two ECoV positive samples. In brief, 3 μg total RNA was extracted from each sample and was used for library construction. Ribosomal (r) RNA was removed by using Epicentre Ribo-zeroTM rRNA Removal Kit (Epicentre, USA). Two sequencing libraries were generated using the rRNA-depleted RNA by NEBNext® UltraTM Directional RNA Library Prep Kit for Illumina® (NEB, USA) following manufacturer’s recommendations. NGS was performed on the Illumina Hiseq 4000 platform and 150 nt paired-end reads were generated. RNA extraction, library construction, and NGS were performed by Novogene (Beijing).

Quality control was performed using Fastp 0.20.0 [12], and clean reads were *de novo* assembled using Trinity 2.5.1 [13]. Blastx search against the GenBank nt (nucleotide) database revealed two contigs from the two samples (30,942 nt for ECoV_2021/464693 and 30,964 nt for ECoV_2020/7881) that shared 97.10% identity with ECoV NC99 (accession no. EF446615, 30,992 nt) at the scale of whole genome. Further analysis showed that 92,022 and 863,418 non-repetitive clean reads were mapped on the assembled contigs, with coverage of 439.98 ± 299.18 (ECoV_2021/464693) (GenBank accession no. OL770366) and 4179.80 ± 968.00 (ECoV_2020/7881) (GenBank accession no. OM937885), respectively. In addition, the assembled viral genome (ECoV_2021/464693) was also confirmed using Sanger sequencing (Table S1), with both sharing 100% sequence identity. To our knowledge, this is the first documented report of ECoV in China.

Multiple sequence alignment of the newly sequenced genome sequences and the representative coronaviruses from the subgenus *Embecovirus* was performed using MAFFT (v7.450) [14] with the E-INS-i algorithm (Data S1) and the novel ECoV genomes were annotated with reference to ECoV NC99. Sequence identities at the scale of whole genome were estimated using Geneious (v2021.0.1). The two novel strains showed 100% sequence identity, and they contained the majority of 5′ and 3′ untranslated regions. Notably, ECoV_2021/464693 shared high sequence identity (between 96% and 97%) with a few representative ECoVs, and had the highest sequence identity of 97.05% with ECoV NC99, the first identified ECoV strain. However, it only shared 85–87% sequence identity with other representative members of the species Betacoronavirus 1 (Figure 1(A)).

**Figure 1.**
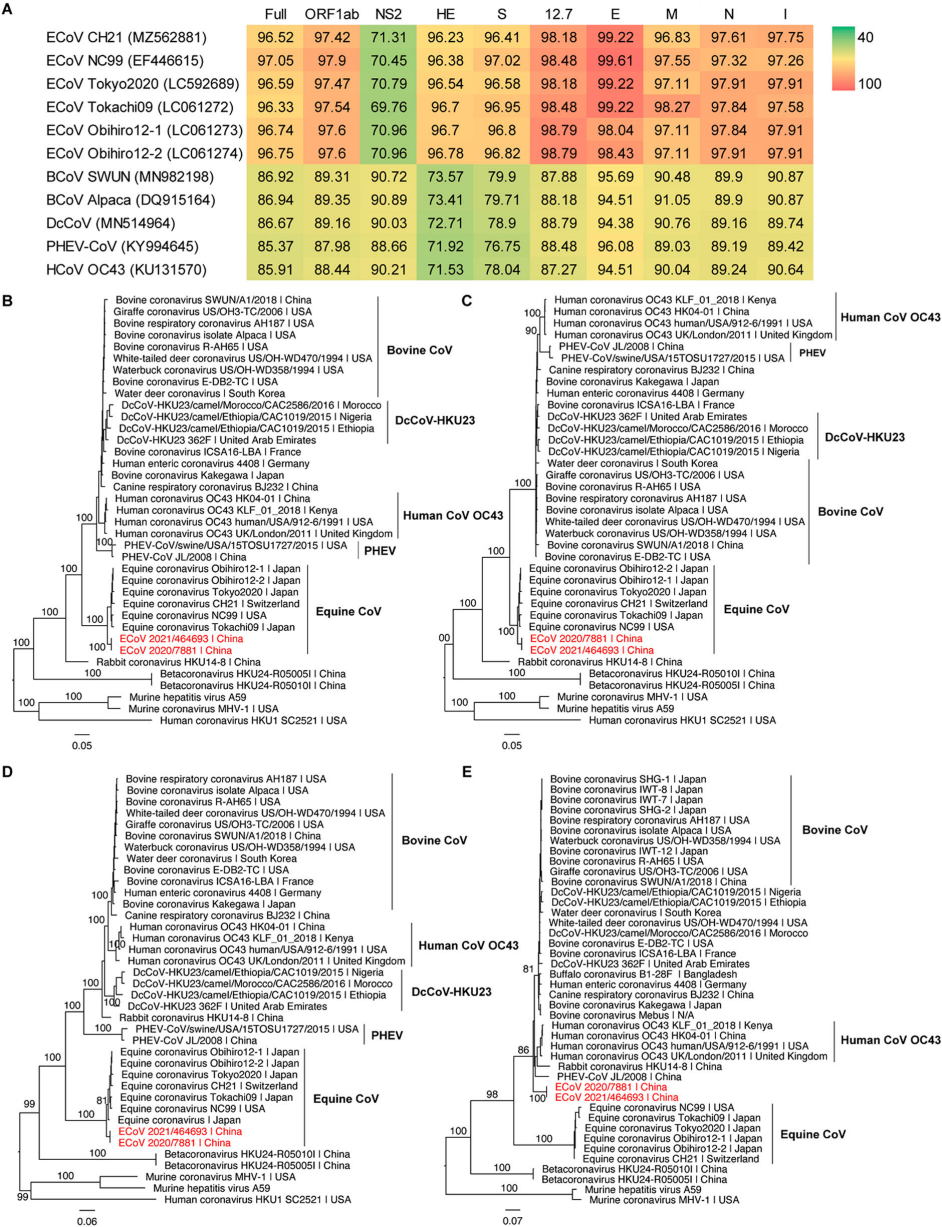
Comparison between the two Chinese ECoV variants and representative members of Betacoronavirus 1. (A) Pairwise sequence identities between ECoV_2021/464693 and related coronaviruses. The degree of sequence similarity is highlighted by colours, with red denoting the highest identities. For the NS2 gene sequences of different length, they were adjusted according to that of ECoV_2021/464693 (582 nt in length). Phylogenetic trees of the full-length virus genome (B), ORF1ab (C), spike gene (D), and the recombinant region (21,523–22,209 nt) (E). Phylogenetic analysis was performed using RAxML (v8.1.6) with 1000 bootstrap replicates and employing the general time reversible nucleotide substitution model and the GAMMA model for rate distribution. The phylogenetic trees were midpoint rooted. The recombinant region was detected to be 21,523–22,209 nt of the ECoV_2021/464693 genome with RDP (v4).

However, previously described ECoVs encode two types of NS2 gene, with the long form of 837 nt (ECoV CH21, NC99, Tokyo2020, and Tokachi09) [15] and the shortened form of 585 nt (ECoV Obihiro12-1 and Obihiro12-2) [16]. The NS2 gene of ECoV_2021/464693 was 582 nt in length and only shared ∼70% sequence identity with other strains of ECoV (Figure 1(A)), suggesting ECoV_2021/464693 to be a potential recombinant. However, these results indicate that the newly reported ECoV strains from China represent a novel variant of ECoV.

To investigate the genetic relationships of the two Chinese strains, we performed phylogenetic analyses with a maximum likelihood approach using RAxML [17]. Phylogenetic analysis of the full-length genome sequences showed that the two novel ECoV strains clustered with representative ECoVs, forming an independent lineage with high bootstrap support (Figure 1(B)). The same phylogenetic relationship was also obtained from the ORF1ab (Figure 1(C)) and the spike (Figure 1(D)) gene trees. Interestingly, the two Chinese ECoV variants formed a separate sub-lineage within the ECoV lineage in these phylogenetic trees (Figure 1(B–D)).

The potential genetic recombination around the NS2 gene region of ECoV_2021/464693 was also supported by different recombination detection methods embedded in the RDP program with *P* values ranging from 1.141 × 10^−98^ for RDP to 4.297 × 10^−21^ for 3Seq. In addition, the breakpoints were estimated to be 21,523–22,209 nt by RDP. Phylogenetic analysis of the potential recombinant region showed that the two Chinese ECoV strains did not cluster with representative ECoVs. Alternatively, they fell within a lineage consisting of bovine CoVs, human coronavirus OC43, and PHEV (Figure 1(E)).

In summary, we identified two ECoV positive samples from donkey foals with diarrhoea from China, from which we successfully obtained two full-length ECoV genomes. Genetic analysis revealed that they represent a novel variant of ECoV and may be a genetic recombinant. To our knowledge, this is the first documented report of ECoV from China, highlighting its expanded geographic range. Therefore, it is necessary to strengthen viral surveillance among horses and donkeys in China to prevent probable CoV outbreaks. In addition, the host spectrum of ECoV and whether ECoV is able to cause human infections warrant further investigation.

## Supplementary Material

Supplemental Material

Supplemental Material
