# Supplementary figures and images for: Identification of a recombinant equine coronavirus in donkey, China
Source: Emerg Microbes Infect. 2022 Apr 4;11(1):1010–3. doi: 10.1080/22221751.2022.2056522 (PMC8986280; doi:10.1080/22221751.2022.2056522)

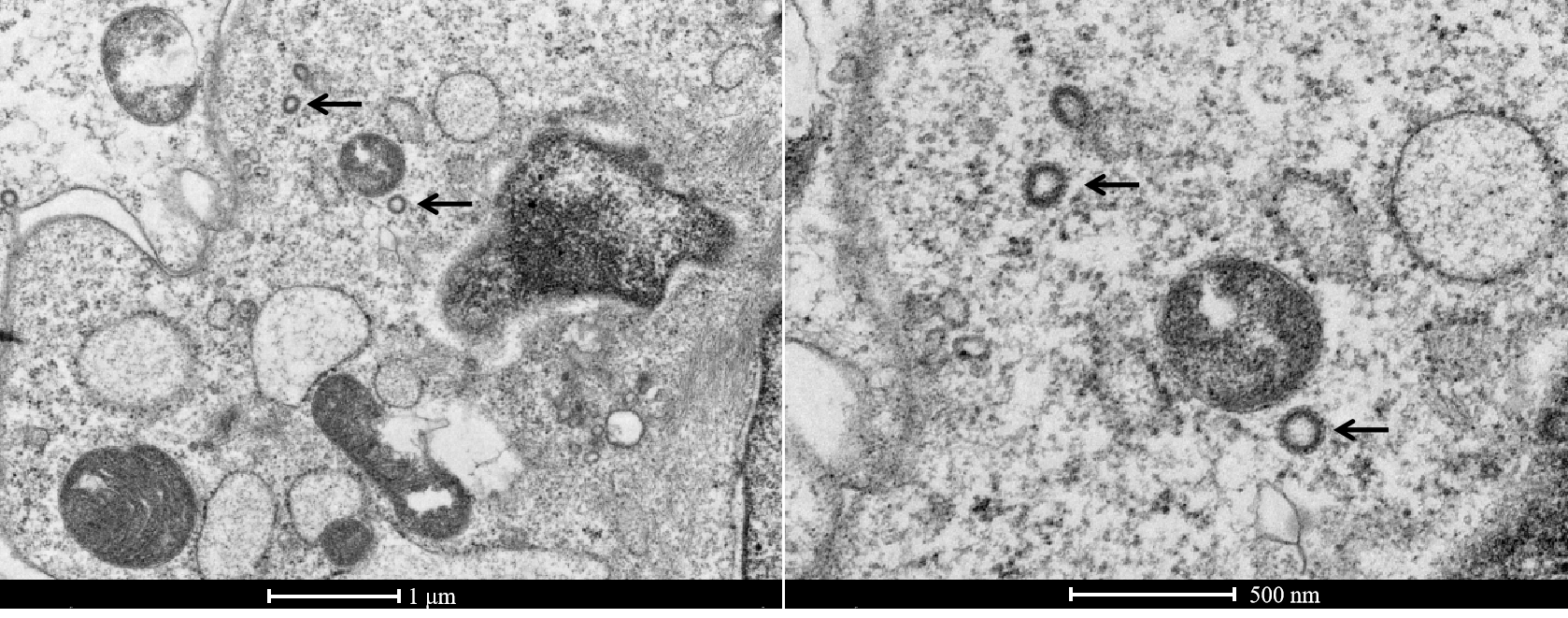

Supplement: Supplemental Material [file TEMI_A_2056522_SM7758.tif]
