# Supplementary material for: Identification of a recombinant equine coronavirus in donkey, China
Source: Emerg Microbes Infect. 2022 Apr 4;11(1):1010–3. doi: 10.1080/22221751.2022.2056522 (PMC8986280; doi:10.1080/22221751.2022.2056522)
Supplement: Supplemental Material [file TEMI_A_2056522_SM7757.docx]

Table S1 Primers used to amplify and sequence the genome of ECoV 2021/464693

| Primer name | Primer Sequence (5’-3’) | Position | Product length (nt) | Gene location |
| --- | --- | --- | --- | --- |
| 1F | GGACCGTGTTATTCAAGATGC | 460-480 | 1614 | ORF1ab:197-21576 |
| 1R | CACATCGCTGCCTCCAAAG | 2055-2073 |  |  |
| 2F | CATTTACTGTTTGTTCAGATGGCTT | 1902-1926 | 1747 |  |
| 2R | TGAGATGTCGCTTGCCATTC | 3629-3648 |  |  |
| 3F | TGTACCTAAAGCAACGCGCA | 3400-3419 | 1554 |  |
| 3R | CACGGCACATATCGAGTGGT | 4934-4953 |  |  |
| 4F | CCTGCAGATTGGCGCATAGT | 4718-4737 | 1571 |  |
| 4R | GCGCTGCAGGATTCAACTTC | 6269-6288 |  |  |
| 5F | TGTTAGGAGGTATGAAAGAGGGT | 6112-6134 | 1634 |  |
| 5R | CTGTAGGCTGAATAGGCCGT | 7726-7745 |  |  |
| 6F | GTAGTACGATCGTCGGTGGC | 7560-7579 | 1548 |  |
| 6R | CGTTCTGCATAAGCCCCTCT | 9088-9107 |  |  |
| 7F | TGCTGATGGAGTGCAGTGTTA | 8953-8973 | 1560 |  |
| 7R | CCAACAGATCCACAAGATCCG | 10492-10512 |  |  |
| 8F | GCCTTTCATGTGACTATGCGT | 10439-10459 | 1644 |  |
| 8R | TGCGTAATCGTCGCAAACTT | 12063-12082 |  |  |
| 9F | GTTGTGGCAGTATTGTAGCACT | 11917-11938 | 1576 |  |
| 9R | CGAGACGGGCATCTACACTC | 13473-13492 |  |  |
| 10F | GTGCAAATTACGCGGCAAGT | 13291-13310 | 1602 |  |
| 10R | TGCCGGTATACACCCACCAT | 14873-14892 |  |  |
| 11F | CCATGGTGGACATTAAGCAGTT | 14807-14828 | 1575 |  |
| 11R | ATCCTGGTGCGTTACACACAT | 16361-16381 |  |  |
| 12F | GAGTGTTGGAGCTTGCGTTG | 16218-16237 | 1607 |  |
| 12R | GCGCTGAATCTACGGTTTGG | 17805-17824 |  |  |
| 13F | GGCGTTACAACACATGAGAGTT | 17647-17668 | 1656 |  |
| 13R | GGCTGCCCTAGAAAAGGGATT | 19282-19302 |  |  |
| 14F | GGTTTGTGTATGTTTTGGAACTGC | 19135-19158 | 1651 |  |
| 14R | GCAAAGTAACTGGCTTCCCG | 20766-20785 |  |  |
| 15F | TCAGTTCATGCTTTGGTGTAACG | 20619-20641 | 1667 | ORF1ab:197-21576  NS2:21586-22167 |
| 15R | GCTTGCTCCATGCATCATCATC | 22264-22285 |  |  |
| 16F | TAGCCACTGGATGA | 22143-22156 | 1605 | NS2:21586-22167  HE:22424-23695 |
| 16R | AAAGCGGTAGGTAG | 23734-23747 |  |  |
| 17F | GTGTATGACCCCTTACCCATT | 23576-23596 | 1635 | HE:22424-23695  S:23710-27801 |
| 17R | TACCTACACAGGTTGCACCA | 25182-25201 |  |  |
| 18F | CTGGAACAGGCGGTATGGTT | 25050-25069 | 1593 | S:23710-27801 |
| 18R | GCAGATGTTGCAGCCAAAGT | 26623-26642 |  |  |
| 19F | AGTAAGGTTGTCTGATGTTGGCT | 26478-26500 | 1584 | S:23710-27801  p12.7:28037-28366 |
| 19R | TCCTCTCAGGTCTCCAGATGT | 28041-28061 |  |  |
| 20F | TAAGACCTGCCTTTGTGGGTT | 27827-27847 | 1584 | p12.7:28037-28366  E:28353-28607  M:28622-29314  N:29324-30664  I:29385-30005 |
| 20R | CTGGTCGGCCCATTTAAGGA | 29391-29410 |  |  |
| 21F | GGCGATAATAGTGGCTTTGCTG | 29201-29222 | 1678 | M:28622-29314  N:29324-30664  I:29385-30005 |
| 21R | TCCCTTATGGCACTTGTCGG | 30859-30878 |  |  |
